# Effect of Electrode Potential on Oxygen Adsorption and Electronic Structure on WC (0001) Surface: An Implicit Solvent DFT Study

**DOI:** 10.3390/ma19061129

**Published:** 2026-03-13

**Authors:** Li Wang, Jiawei Wei, Chaofan Yin, Ying Liu, Fan Bai, Binbin Dong

**Affiliations:** 1 Henan Key Laboratory of Green Building Materials Manufacturing and Intelligent Equipment, Luoyang Institute of Science and Technology, Luoyang 471023, China; weijiaweicera@126.com (J.W.); ycfwust@163.com (C.Y.); yingliu_77@163.com (Y.L.); 13137905128@163.com (F.B.); 2 School of Materials Science and Engineering, Jiangxi University of Science and Technology, Ganzhou 341000, China

**Keywords:** tungsten carbide, electrocatalysis, adsorb, implicit solvent model, density functional theory

## Abstract

To facilitate the next generation of renewable energy devices, it is important to engineer oxygen reduction reaction (ORR) catalysts that balance efficiency and production costs. This work examines oxygen adsorption on the WC (0001) surface as a function of electrode potential, utilizing DFT simulations with an implicit solvent environment. The results demonstrate that electrode potential significantly influences oxygen adsorption energy and electronic structure. Among the adsorption sites examined, the top site exhibits the highest stability across the entire potential range. The observed reduction in adsorption energy at lower potentials is attributed to the d-band center moving further from the Fermi energy, which weakens C–O orbital interactions, as revealed by DOS and COHP analyses. Our results demonstrate the crucial role of electrochemical conditions in modulating catalytic behavior and provide valuable insights for optimizing tungsten carbide (WC)-based electrocatalysts for ORR applications.

## 1. Introduction

The rapid development of renewable energy technologies has necessitated the rapid development of advanced technologies for energy conversion and storage, such as fuel cells and metal–air batteries, which rely on electrochemical reactions for energy generation [1,2]. Among the key reactions in these systems, the oxygen reduction reaction (ORR) is of paramount importance due to its role in the cathode process, where oxygen is reduced to form water [3]. However, the sluggish kinetics of ORR remain a significant challenge, making the engineering of high-performance, economically viable electrocatalysts a priority [4,5]. Platinum (Pt)-based catalysts have been extensively used due to their superior catalytic performance, but their high cost, limited availability, and poor sustainability have spurred the search for alternative materials [6,7].

Tungsten-based materials, particularly tungsten carbides (WC), have garnered significant attention for oxygen reduction applications, owing to their high terrestrial abundance and low material costs, high stability, and tunable electronic properties [8,9]. The unique characteristics of WC, including its Pt-like electronic structure and robust mechanical properties, make it a potential substitute for noble metals in electrocatalytic processes [10]. As a key member of the 5d transition metal family, WC exhibits strong metal-oxygen interactions, making it a viable candidate for oxygen-related reactions [11]. Additionally, the electrocatalytic efficacy of WC exhibits a strong sensitivity to its surface termination, including the nature of active sites and the ability to modulate these sites via surface modifications [12].

While the intrinsic properties of WC-based catalysts have been explored in numerous studies, the role of solvents and electrochemical environments, including electrode potentials, in modulating the electrocatalytic activity remains underexplored [13]. Traditional theoretical models often assume idealized conditions such as vacuum and charge neutrality, which fail to account for the complex interactions occurring in real electrochemical environments [14]. To address this gap, the introduction of implicit solvent models into DFT calculations offers a more accurate representation of the electrochemical interface, including solvent effects and the influence of applied electrode potentials [15,16].

In this study, we investigate the adsorption behavior of oxygen on the WC (0001) surface under varying electrode potentials using the implicit solvent model. This approach allows us to evaluate the impact of solvent interactions and electrochemical conditions on the adsorption energies and stability of oxygen intermediates, which are crucial for the ORR mechanism [17]. By combining computational methods with implicit solvent modeling, we aim to provide a deeper understanding of the catalytic performance of WC-based materials in real-world electrochemical environments, which informs the development of next-generation catalytic systems for efficient energy conversion applications.

## 2. Materials and Methods

Density functional theory (DFT) calculations were carried out using the Materials Studio package (Materials Studio 2023). The exchange-correlation interaction was treated by the generalized gradient approximation (GGA) with the Perdew–Burke–Ernzerhof (PBE) functional [18,19].

The PBE functional was employed as it reliably describes the electronic trends of metallic WC, where the delocalized nature of 5d electrons reduces the self-interaction errors typically found in more localized systems. A plane-wave basis set with a kinetic energy cutoff of 520 eV was adopted. We sampled the Brillouin zone using Monkhorst–Pack grids of 5 × 5 × 5 for the bulk and 5 × 5 × 1 for the slab models. Structural relaxation was performed until the convergence tolerances for energy and force reached 10^−5^ eV and 0.02 eV/Å, respectively. The WC (0001) surface was simulated by a (3 × 3) supercell containing five atomic layers separated by a 15 Å vacuum region. The weak van der Waals interactions were accounted for using the empirical Grimme’s DFT-D3 correction scheme. During optimization, the bottom three layers were constrained to bulk positions, while the upper layers were allowed to relax.

Solvation effects were incorporated using the implicit solvation scheme implemented in the VASP solcode (vasp.6.3.2) [20], which integrates a continuum dielectric medium into the plane-wave DFT framework. To mimic the aqueous electrolyte, the relative dielectric constant was set to 78.4. The electrode potential was modulated by varying the total electron count, with the counter-charge distribution described by the linearized Poisson-Boltzmann equation (Gouy–Chapman model). A Debye screening length of 3.04 Å was adopted to characterize the ionic strength. Consequently, the grand canonical energy of the interface is defined as [21]:

(1) $$E=E_{VASP}+e\emptyset_{elyte}∆q-\left(\mu_{e}+e\emptyset_{elyte}\right)∆q=E_{VASP}-E_{F}∆q$$

Here, E_VASP_ denotes the total energy computed by VASPsol, and $\emptyset_{e}$ represents the electrostatic potential reference in the bulk electrolyte. Under the Kohn-Sham-Mermin formalism, the electron chemical potential is equated to the Fermi level (E_F_). The net charge is given by Δq (positive for electron accumulation, negative for depletion). The conversion between E_F_ and the applied potential U (relative to the standard hydrogen electrode, SHE) follows the relation [16]:

(2) $$U\left(V\;vs.SHE\right)=-4.44\;V-(E_{F}+E_{Fermishift})/e$$

where E_Fermishift_ accounts for the Fermi level correction term applied by VASPsol. The adsorption energy is thus defined as:

(3) $$E_{ads}\;=\;E_{post\text{-}adsorption}\;-\;E_{pre\text{-}adsorption}$$

where *E_post-adsorption_* and *E_Pre-adsorption_* denote the total energies of the pristine slab and the adsorbate-substrate complex, respectively. By fitting the total energy to a quadratic function, we derived the linear dependence of adsorption energy on electrode potential. The VASPKIT [22] code was employed to process and analyze data from VASP calculations. For visualization of the structural models and charge distributions, VESTA (VESTA. 3. 90. 5a) [23] software was utilized. Crystal orbital Hamilton population (COHP) analyses were conducted using LOBSTER [24,25], which was also used to evaluate the integrated crystal orbital Hamilton population (ICOHP) to quantify the strength of the chemical bonds.

## 3. Results and Discussion

### 3.1. Adsorption Energy and Configuration

**Figure 1:**
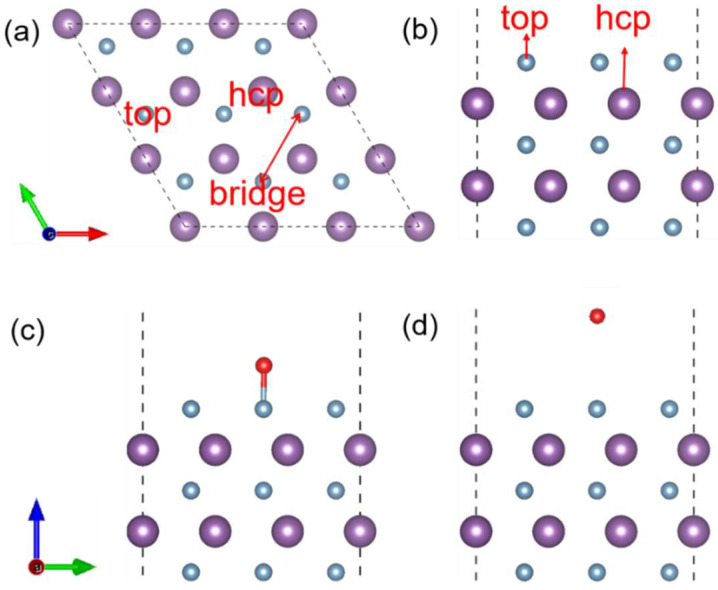
(**a**,**b**) Schematic diagram of different adsorption sites on the surface of WC (0001), and structural models of (**c**) post-adsorption and (**d**) pre-adsorption.

Based on the analysis of adsorption behavior across different sites, three high-symmetry positions (top, hcp, and bridge) were examined for pristine WC (0001) (Figure 1a,b). Notably, the bridge site exhibited unstable adsorption, whereas a comparison of oxygen adsorption energies under both vacuum and neutral conditions revealed that the top and hcp site provides the most favorable environment. Thus, these sites were chosen as the initial adsorption geometries for VASPsol simulations under various electrochemical potentials.

**Figure 2:**
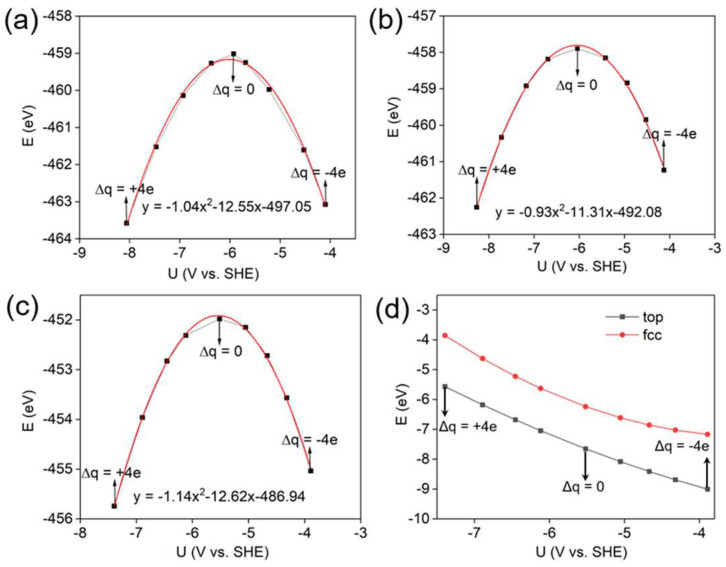
Total energy (black) and polynomial fitting (red) for oxygen on the WC (0001) surface at (**a**) top-site, (**b**) fcc-site, (**c**) before adsorption and (**d**) adsorption energies at different electrode potentials (U). The formula in the figure is the corresponding quadratic function.

To elucidate how the electrochemical potential modulates the adsorption strength, two distinct models were constructed representing the states before and after oxygen adsorption on the WC (0001) surface (Figure 1c,d). For each model, calculations were carried out over a charge range from −4e to +4e, with increments of +1e. Based on Equations (1) and (2), the system’s potential and energy were determined to span from −3.63 to −7.75 V versus SHE by either removing or adding electrons. As illustrated in Figure 2a–c, the E-U data points fit well to a quadratic function described by

(4) $$E\left(U\right)=-\frac{1}{2}C{\left(U-U_{PZC}\right)}^{2}+E_{PZC}$$

where U*_PZC_* denotes the potential of zero charge, E*_PZC_* is the corresponding energy at U*_PZC_*, and C represents the surface capacitance. Based on Equation (3) for adsorption energy, the dependence of adsorption energy on electrode potential for oxygen atoms at various adsorption sites on the WC (0001) surface can be derived. As shown in Figure 2d, the adsorption energy increases with decreasing electrode potential, and the top site consistently exhibits more stable adsorption across the entire potential range.

### 3.2. Electronic Structure of Oxygen Adsorption on the WC (0001) Surface

To further elucidate the mechanism of oxygen adsorption on the WC (0001) surface, we performed a comprehensive electronic structure analysis focusing particularly on the top site, which showed the highest stability among the investigated sites.

**Figure 3:**
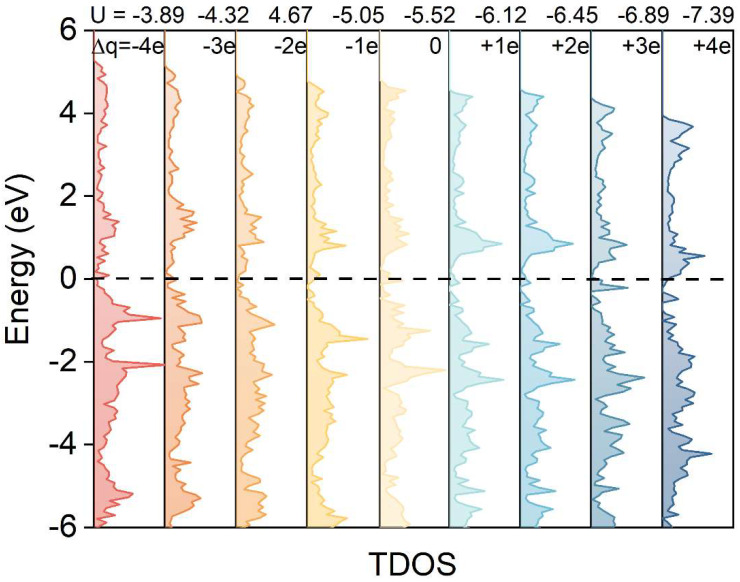
TDOS plots of adsorbed oxygen on the WC (0001) surface at different electrode potentials.

**Figure 4:**
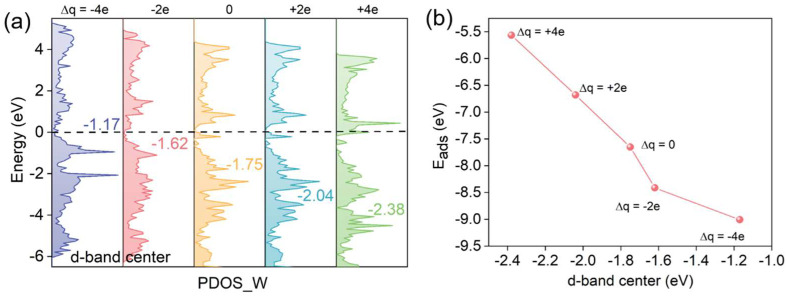
(**a**) PDOS plots of elemental tungsten. The d-band center was marked in the figure. (**b**) Plot of adsorption energy versus d-band center.

Figure 3 shows the total density of states (TDOS) for oxygen adsorbed on the WC (0001) surface at various electrode potentials. Within the charge range of −4e to +4e, the electrode potential varies from −3.89 V to −7.39 V. The introduction of extra electrons causes the Fermi level to shift upward toward the conduction band, approaching or even entering it. These findings demonstrate that electrode potential substantially influences both the adsorption characteristics and electronic configuration of oxygen on the WC (0001) surface. Additionally, the PDOS of elemental tungsten exhibits a similar trend. Notably, when electrons are introduced into the system, as the electrode potential decreases, the d-band center gradually shifts away from the Fermi level (from −1.17 to −2.38 eV), while the adsorption energy decreases linearly (Figure 4a). The quantitative relationship between the electrode potential (U) and the d-band center on the WC (0001) surface is primarily governed by the change in surface charge density. As the potential decreases (becomes more negative), the surface accumulates excess electrons, leading to a progressive filling of the d-states and an upward shift in the Fermi level. This observation is in line with the established d-band center theory, which states that the further the d-band center is from the Fermi level, the more antibonding orbitals are filled, thereby weakening bond stability and reducing adsorption strength [26,27].

**Figure 5:**
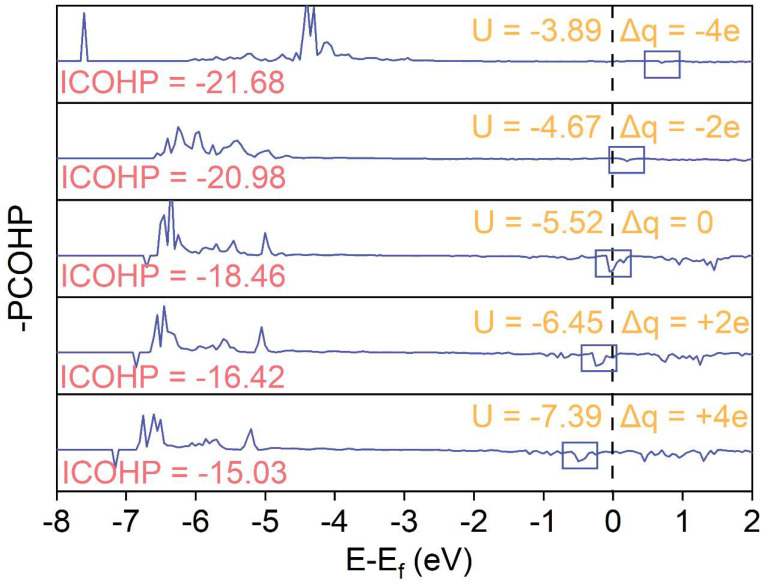
Average pCOHP and integrated COHP (ICOHP) for the C-O bond at different electrode potentials.

The Crystal Orbital Hamilton Population (COHP) method further decomposes the total density of states into contributions from bonding and antibonding interactions, thereby providing an intuitive description of the strength and nature of the chemical bonds between oxygen atoms and carbon atoms on the WC surface [28]. As shown in Figure 5, as the electrode potential decreases, the ICOHP value gradually becomes positive, indicating a weakening of the C-O bond interactions, which results in a reduction in the adsorption energy and less stable adsorption; moreover, when electrons are added to the system, the peaks near the Fermi level shift to the left, suggesting that the high-energy antibonding orbitals are increasingly filled, further weakening the adsorption stability [29].

## 4. Conclusions

This study provides a comprehensive investigation of oxygen adsorption on WC (0001) surfaces under varying electrode potentials using DFT combined with an implicit solvent model. The results show that decreasing electrode potential weakens C–O bond interactions, as evidenced by shifts in the d-band center and a reduction in adsorption energy. Among the adsorption sites analyzed, the top site exhibits the most stable adsorption across different potentials. The analysis of electronic structure, including COHP and DOS, confirms that changes in electrode potential modulate antibonding orbital occupancy, thereby influencing adsorption strength and stability. These insights offer a theoretical basis for enhancing the electrocatalytic activity of WC-based materials and provide valuable guidelines for the design of efficient, non-noble metal catalysts for the oxygen reduction reaction in energy conversion systems.

## Data Availability

The original contributions presented in this study are included in the article. Further inquiries can be directed to the corresponding authors.
